# What role for cardiac imaging in chronic coronary syndromes: review of the literature in light of the latest recommendations

**DOI:** 10.1093/ehjimp/qyaf112

**Published:** 2025-08-20

**Authors:** David Sulman, Stéphane Manzo-Silberman

**Affiliations:** Sorbonne University, Institute of Cardiology-Hôpital Pitié-Salpêtrière (AP-HP), ACTION Study Group, Paris, France; Sorbonne University, Institute of Cardiology-Hôpital Pitié-Salpêtrière (AP-HP), ACTION Study Group, Paris, France; Chair of the EAPCI Patient Advocacy Committee, EAPCI 2024-2026

**Keywords:** chronic coronary syndrome, coronary artery disease, non-Invasive imaging, coronary computed tomography angiography, cardiovascular magnetic resonance, high-Risk plaque

## Abstract

The 2019 ESC guidelines redefined stable coronary artery disease as chronic coronary syndrome (CCS), highlighting the dynamic nature of this disease. This condition is characterized by the gradual accumulation of atherosclerotic plaques in the epicardial coronary arteries. CCS can result in myocardial ischaemia due to supply–demand mismatch, often triggered by physical or emotional stress. The clinical course may be abruptly interrupted by plaque rupture or erosion, leading to acute coronary syndromes. Revolutionary advances in non-invasive imaging have transformed the chronic coronary syndrome diagnosis algorithm and management. Coronary computed tomography angiography provides detailed anatomical insights, identifying high-risk plaques with features like low attenuation and positive remodelling, as evidenced by SCOT-HEART, which reported reduced coronary events (HR: 0.59, *P* = 0.004). Stress echocardiography may detect ischaemia-induced wall motion abnormalities (sensitivity, 85–95%), while cardiovascular magnetic resonance is paramount in functional assessment, offering 81–86% sensitivity/specificity and detecting microvascular dysfunction via perfusion and late gadolinium enhancement. Nuclear imaging (SPECT/PET) enhances ischaemia detection, with PET’s myocardial flow reserve improving prognostic accuracy (sensitivity 90%, specificity 88%). AI-driven innovations, such as CT-derived fractional flow reserve, automate plaque quantification and may reduce in the future unnecessary invasive angiographies by 19–25% (*P* = 0.01), while dynamic CT myocardial perfusion integrates anatomical and hemodynamic data, boosting diagnostic accuracy (87%). These advancements enable precise risk stratification and a personalized multimodal imaging approach, based on pre-test likelihood. It also increases the risk of unsustainable costs for society, repeated radiation exposure throughout a patient's life, and raises the question of actual limited benefits from revascularization in low-risk patients.

## Graphical Abstract

**Graphical Abstract:**
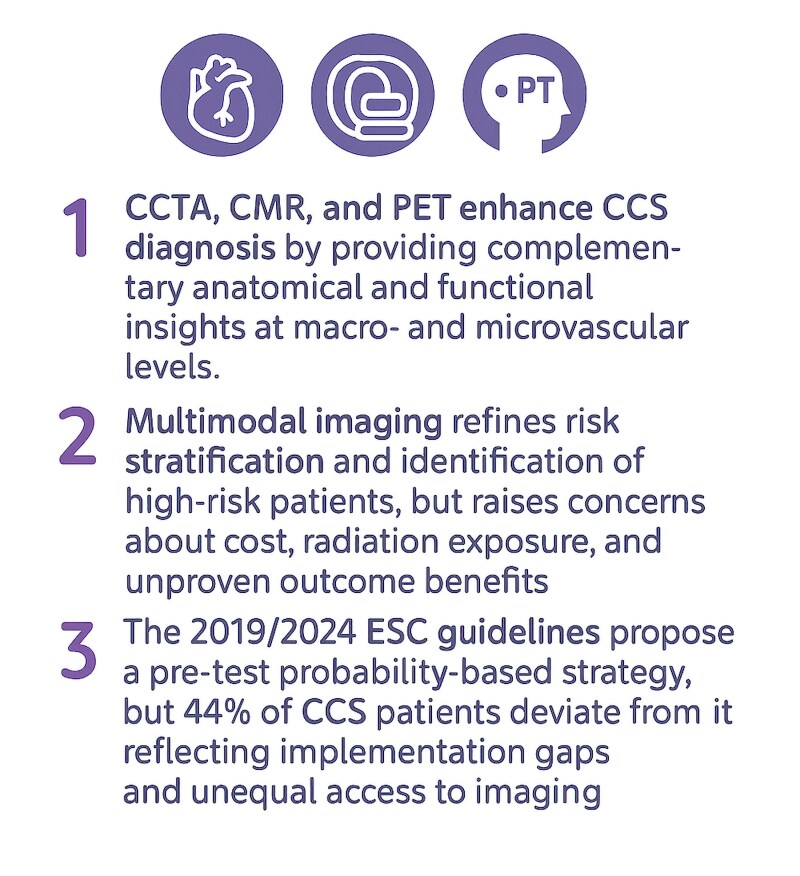

## Introduction

In 2019, the ESC guidelines defined the concept of chronic coronary syndrome (CCS),^1^ replacing the term of obsolete stable coronary artery disease (CAD), to better categorize the different patterns of CAD during its evolution, particularly before or after an acute coronary syndrome (ACS). CAD is described as a continuous, progressive pathological process that is characterized by the accumulation of atherosclerotic plaques in epicardial arteries, whether obstructive or non-obstructive.^2^

These alterations can cause a transient, typically reversible imbalance between myocardial demand and blood flow, leading to ischaemia, often triggered by physical or emotional stress.^3^ Symptoms may manifest as angina, dyspnoea, or may be entirely absent.

CCS can remain stable but may destabilize, with ACS often triggered by vulnerable plaque rupture. A deeper understanding of CCS pathophysiology drives a complex diagnostic algorithm, integrating macro- and microvascular abnormalities. CCS can now be diagnosed at a microvascular level without epicardial disease via vasospasm, myocardial bridging, or microvascular dysfunction (INOCA).^4^

Advances in imaging have driven this paradigm shift and modified our diagnostic algorithms, leading to increased detection of CAD, particularly among patients with previously unrecognized microvascular symptoms. While it remains impossible to predict with certainty which plaques will rupture and cause an ACS, anatomical and functional imaging enable better characterization of patients. Imagery can now identify high-risk plaques or population, allowing for a more accurate, non-invasive assessment of cardiovascular event risk.

**Figure 1:**
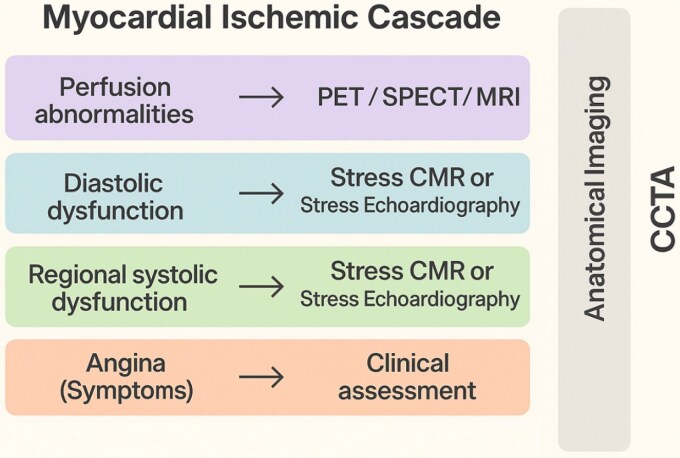
Summary of imaging modalities in the coronary ischaemic cascade.

Each imaging modality detects ischaemic cascade defects (*Figure 1*), requiring careful selection based on its specific strengths and limitations for each patient.

In complement to the previous review focusing on intracoronary imaging,^5^ this review will explore the various non-invasive anatomical and functional imaging modalities and evidence supporting their use in CCS.

### Anatomical imaging techniques

#### Coronary computed tomography angiography (CCTA)

Coronary computed tomography angiography (CCTA) is an appealing imaging technique, enabling simultaneous analysis of the vessel wall and lumen without the need for invasive angiography, which carries a minimal but inherent risk of complications.

There has been a significant increase in the use of CCTA worldwide and in Europe over the past decade. The EURECA registry reported that CCTA was used in 22% of patients with chronic coronary syndrome between 2019 and 2020 in 24 ESC member countries, with a striking 2098% increase in CCTA prescriptions in Denmark, for instance.^6,7^ In patients with low-to-moderate risk, CCTA provides precise evaluation of epicardial coronary tree, with short acquisition and improved resolution due to the advent of multi-slice scanners.

CCTA has gained prominence following positive studies summarized in *Table 1*. The SCOT-HEART study,^8,9^ a landmark open-label trial, demonstrated its benefits. Patients were randomly assigned (1:1) to standard care plus CCTA or standard care alone. Nearly half of the participants had a baseline clinical diagnosis of coronary heart disease, and over a third had anginal symptoms due to coronary heart disease. At 6 weeks, CCTA reclassified the diagnosis of coronary heart disease in 27% patients and the diagnosis of angina due to coronary heart disease in 23% patients. Coronary deaths and non-fatal myocardial infarctions (MI) were reduced in the CCTA group over 5 years (HR: 0.59; 95% CI: 0.41–0.84; *P* = 0.004) with better risk stratification and a good negative predictive value. CCTA group was also more likely to receive preventive therapies,^8,9^ with an odds ratio of 1.17 (95% CI: 1.01–1.36, *P* = 0.034).

**Table 1:** Studies providing positive impact of CCTA

Study Name	Year	Comparison	Findings
**PROMISE** ^10^	2015	CCTA vs. functional stress tests	Similar outcomes at 25 months; CCTA more accurate, especially in diabetics (HR: 0.38; 95% CI: 0.18–0.79; *P* = 0.01).
**SCOT-HEART** ^11^	2018	−CCTA + standard care vs. standard care alone	Reduced coronary deaths and non-fatal MI over 5 years (HR: 0.59; 95% CI: 0.41–0.84; *P* = 0.004); better risk stratification. Low-attenuation plaque burden tripled MI risk, independent of risk scores, CACS, or stenosis severity.
**DISCHARGE^12^**	2022	CCTA vs. invasive coronary angiography (ICA)	Fewer unnecessary invasive procedures; similar outcomes over 3.5 years.

The rates of revascularization procedures were similar between the CCTA group and the standard care group (HR: 1.00, *P* = 0.99). In patients with suspected angina due to CAD, CCTA clarifies the diagnosis, enables targeted interventions, and may reduce the future risk of MI. Similarly, other studies^11,13^ compared CCTA to non-invasive functional tests in CCS, showing similar outcomes at 25 months with CCTA being more accurate, especially in diabetic patients (HR: 0.38; 95% CI: 0.18–0.79; *P* = 0.01).^3^ Furthermore, CCTA compared to invasive angiography in CCS showed similar cardiovascular event rates but with the benefit of reduced coronary angiography procedures.^12^

**Figure 2:**
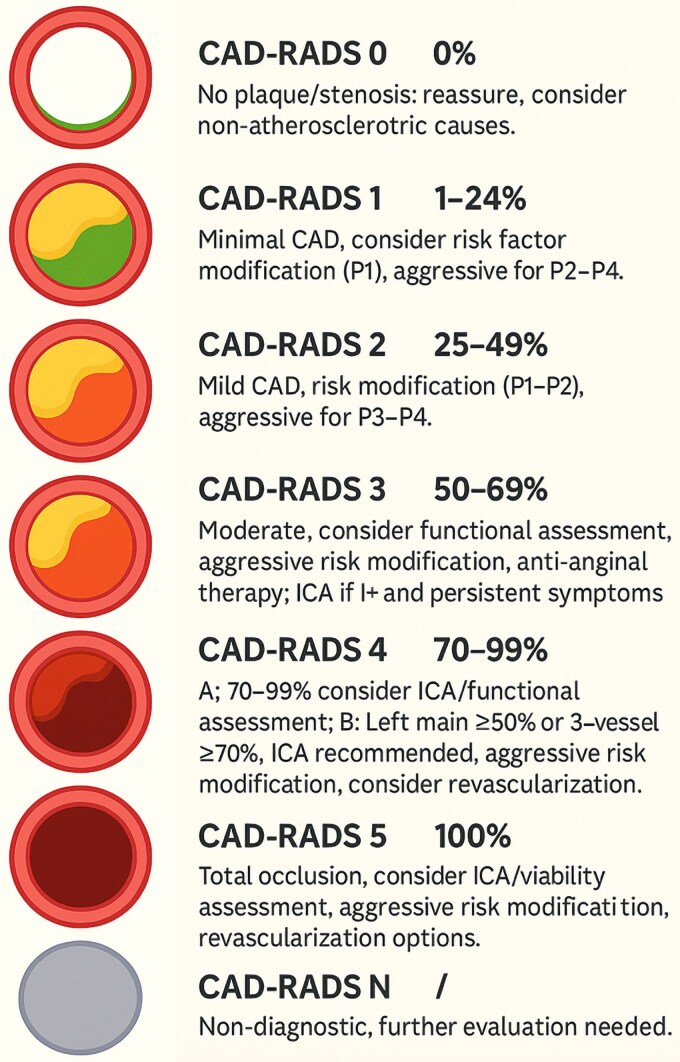
CADRADS 2.0 radiology classification.^14^

The improvement of the CAD-RADS classification^14^ has contributed to better plaque description and homogenized analysis practices. It is currently the recommended method for quantifying stenoses and providing clear management guidance (*Figure 2*). It enables precise lesion characterization, especially high-risk plaques, but is less suited for follow-up imaging than the Segment Stenosis Score (SSS) and Segment Involvement Score (SIS).^15^

CCTA identifies vulnerable high-risk plaque (HRP) features that strongly predict MI, beyond traditional risk factors, coronary artery calcium score (CACS), or stenosis severity. Similarly, Perivascular Fat Attenuation Index (FAI), a biomarker of coronary inflammation measured via perivascular adipose tissue attenuation on CCTA (range: −30 to −190 HU), showed a significant link to increased cardiovascular mortality over more than 7 years, with risk rising as more vessels were concerned.^16^

Even in young patients with premature CAD, there is a higher prevalence of HRP features, including spotty calcification, positive remodelling, low attenuation, and napkin-ring sign. These features are more frequent in patients with premature CAD compared to matched controls.^17^ The presence of these HRP is associated with an increased risk of ACS, with positive remodelling and low-attenuation plaques being particularly predictive of future adverse events.^18^

**Figure 3:**
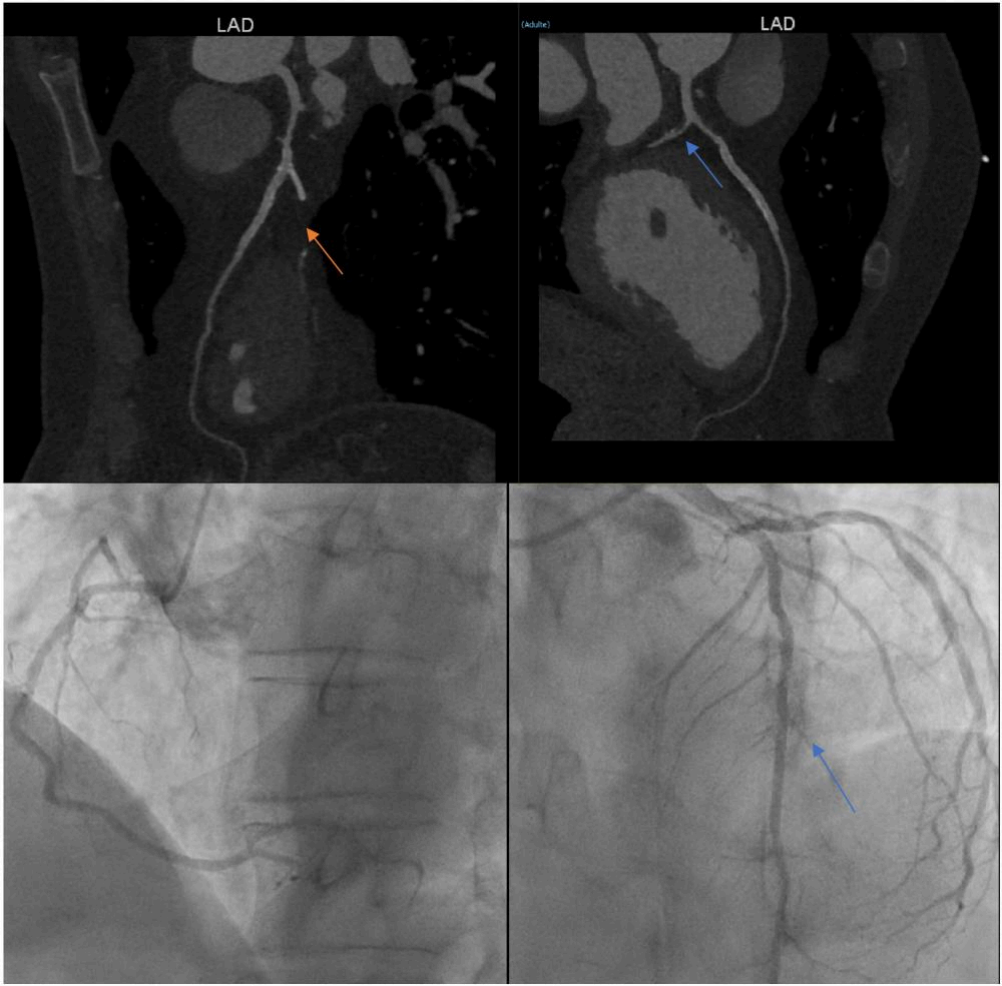
CCTA and angiogram in a 55-year-old patient with recurrent chest pain showing little diagonal occlusion (top left arrow) and high-risk plaque of circumflex and LAD (top right arrow).

We present, for illustration, the case of a 55-year-old man with multiple cardiovascular risk factors and a history of multivessel PCI for STEMI (February 2023), who presented with recurrent chest pain. CCTA showed patent LAD and RCA stents, suspicion of occlusion of the distal first diagonal branch (top left arrow), and high-risk LAD and circumflex plaques with moderate-to-intermediate stenoses (low Hounsfield units, spotty calcium—top right arrow) (CAD-RADS 5/P2/HRP/S) (*Figure 3* and Supplementary data online, *Video 1*). Troponin levels were normal. A coronary angiogram was performed (*Figure 3*), and the initial decision was to proceed with medical treatment only, consisting of high-dose statins, ezetimibe, and intensification of antianginal therapy, in view of the high-risk plaque profile and the new event.

Zanon *et al*.^19^ also showed that adding an adapted ultra-low-dose CT protocol to CCTA allowed incidental detection of pulmonary nodules in 41% of patients, with a 3% diagnostic yield for lung cancer, particularly due to the often-smoking population screened.

Limitations of CCTA include challenges in patients with high or very high cardiovascular risk, where extensive calcifications (e.g. in long-standing diabetics or those with chronic kidney disease) may cause artefacts. Also, the use of iodinated contrast, although now limited to what is necessary, carries a rare but potentially serious risk of allergic reaction and acute kidney injury.^20^ Moreover, high heart rates may also affect acquisition, though this is less problematic with modern scanners.

It is also important to highlight that the CACS in CCS has no role as CAD diagnostic tool. In fact, CACS is limited to coronary calcium quantification and is useful to enhance cardiovascular risk stratification in asymptomatic patients over 50 years. A meta-analysis of 34 000 asymptomatic patients showed that high CACS was strongly and independently associated with an increased risk of major adverse cardiac events (MACE), with risk increasing as the CACS increases.^21^ Coronary calcium is rarer in younger adults (<45–50 years). Nevertheless, its diagnostic sensitivity for obstructive CAD is lower in patients under 50, and a non-negligible proportion of younger symptomatic patients with obstructive CAD will have a CAC score of 0.^22,23^

CCTA/CACS may sometimes overestimate coronary stenosis, and diagnosis can be refined through a multimodal approach. For example, a 48-year-old man with dyslipidaemia, hypertension, and ankylosing spondylitis presented with exertional chest pain. CCTA showed ‘severe, calcified and significant triple-vessel disease’ (CAD-RADS 4A P3), including near-occlusive stenoses and a dissected plaque. However, angiography revealed overestimation by CCTA. With no critical epicardial stenosis except for a small diagonal. Additional microvascular angina was also suspected, and symptoms quickly improved with optimized treatment using calcium inhibitors (see Supplementary data online, *video 2*) without any PCI.

### Functional imaging techniques

#### Stress echocardiography (SE)

Stress echocardiography can be performed with physical exercise (treadmill or bicycle) or with pharmacological agents (dobutamine, adenosine) in patients unable to exercise. It allows the detection of wall motion abnormalities induced by ischaemia or the evaluation of segmental viability, which are sensitive and specific markers of obstructive CAD. Its sensitivity and specificity have been reported in several studies or recommendations, such as a meta-analysis of 44 studies, around 85–95% and a specificity of 77–96%^21,24^ for the detection of stenoses >50%.

It also allows prognostic stratification.^25^ A normal stress echocardiography suggests a favourable prognosis but does not completely exclude coronary artery disease, with an event rate of 0.9%. Moreover, it is possible to evaluate the left ventricular contractile reserve and sometimes the coronary flow reserve, providing additional information on the disease severity.^26^

One can mention the contribution of echocardiography using Doppler evaluation of the LAD, but this represents a difficult evaluation with significant operator variability.^27^ This non-invasive modality presents limitations notably.

The image quality (even if sometimes partially improved by contrast), the variable response to physical stress or inotropes, the subjective operator interpretation, the variability of sensitivity and specificity, and the technical challenges related to rapid image acquisition.

### Cardiovascular magnetic resonance (CMR)

Morphological and stress cardiovascular magnetic resonance (CMR) have the major advantage of being a non-irradiating examination.

A meta-analysis evaluated the diagnostic and prognostic value of stress CMR in patients with stable chest pain. This study observed that stress CMR had a sensitivity of 81% and a specificity of 86% for detecting functionally significant obstructive CAD.^28^

The CE-MARC study demonstrated multiparametric CMR’s superiority over SPECT for detecting single- or multivessel CAD, with 86.5% sensitivity and 83.4% specificity, using invasive coronary angiography as the reference.^29^

The CE-MARC 2 trial showed CMR- or SPECT-guided strategies reduced unnecessary angiographies compared to standard care, without increasing MACE.^30^ Another meta-analysis confirmed these results with similar sensitivity and specificity for stress perfusion CMR in detecting CAD, this time compared to invasive FFR.^31^

Its certain advantage is the acquisition of multiple phases, notably cine sequence, which allows analysis of left ventricular/right ventricular kinetics. Gadolinium perfusion assesses macrovascular coronary perfusion but also potentially microvascular perfusion, as well as late enhancement studies.

Perfusion is studied in stress CMR, preferably pharmacologically (dobutamine or vasodilators such as Regadenoson). It allows the detection of myocardial ischaemia with good spatial resolution, identifying subendocardial perfusion defects.

Dobutamine assesses contractile reserve but may reduce image quality and raise arrhythmia risk, while vasodilators induce hyperaemia for perfusion sequences to detect hypoperfused segments.

The automation of acquisition now allows for assessing myocardial blood flow (MBF) and myocardial perfusion reserve (MPR) maps, improving the detection of ischaemia, multivessel CAD, and microvascular dysfunction compared to visual operator assessment.

Therefore, stress CMR represents an efficient and non-irradiating tool for CCS by detecting ischaemia and assessing the extent of CAD. CMR also allows the detection of fibrosis and myocardial scars via the measurement of Late Gadolinium Enhancement (LGE) by CMR. A sequence of inversion-recovery is used ∼10 min after gadolinium injection to unveil fibrotic areas.

Phase-sensitive inversion recovery techniques may help to improve differentiation between healthy and pathological tissue. This examination sequence has been validated against histology with excellent sensitivity.^32^

T1 mapping and extracellular volume (ECV) fraction identify ischaemic regions by detecting elevated native T1 values (replacement fibrosis) or reduced values (fatty metaplasia). Elevated ECV in infarcted areas enhances quantification of myocardial fibrosis. LGE also has prognostic value.

The transmural or non-transmural nature of LGE is crucial for predicting functional recovery after revascularization. In fact, 90% of myocardial segments with LGE >50% transmurally do not improve post-revascularization.^33^ An LGE ≤50% indicates limited scar and preserved viability, while >50% reflects extensive damage with poor prognosis. Each 5% increase in infarct size (measured by LGE) is associated with a 19% increase in 1-year mortality. The extent of LGE also correlates with higher rates of MACE and potentially fatal arrhythmias, making it a key element in ischaemic heart disease assessment.^34^ Consequently, identifying viable (≤50%) vs. non-viable (>50%) myocardium via LGE helps guide revascularization decisions.^35^

The limitations of CMR include its limited accessibility, claustrophobia, long acquisition time, interference with metallic prostheses, and difficulties in interpretation with a learning curve.

**Figure 4:**
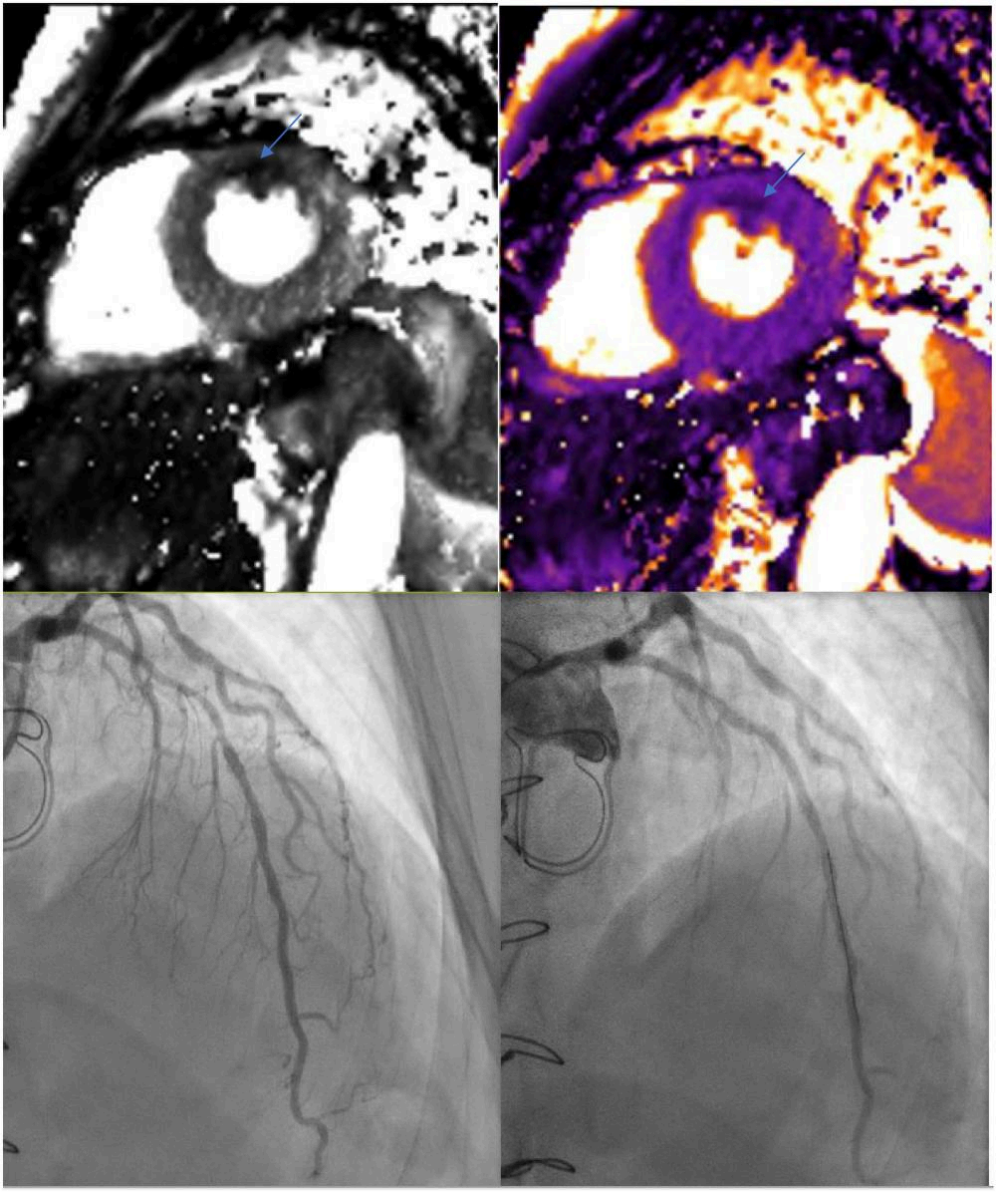
Multimodal imaging in a 75-year-old post-Bentall patient using stress MRI showing new anterior ischaemia (arrow) beyond prior necrosis due to evolving and ischaemic long LAD lesion, successfully treated by PCI.

We present the case of a 75-year-old man with a history of a Bentall procedure (2018) for annulo-aortic ectasia, who at that time had an isolated antero-basal necrosis with >50% transmurality and a CCTA which showed mid-LAD intermediate plaque treated medically. He recently reported the onset of new anginal pain, with a multimodal approach using stress MRI showing the appearance of a new anterior ischaemia (3/17 segments) (*Figure 4* and Supplementary data online, *video 3*), extending beyond the previously known necrotic area. Coronary angiography confirmed the lesion, and he subsequently underwent PCI of the LAD, along with intensification of statin therapy and improved blood pressure control.

### Nuclear imaging (SPECT, PET)

Single-photon emission computed tomography (SPECT) is widely used for functional screening of patients suspected of CCS. The detection of necrosis and/or extensive perfusion defects helps identify high-risk patients for events and/or those potentially having significant single- or multivessel CAD.

A meta-analysis of 114 SPECT studies in patients with suspected or established CAD showed moderate sensitivity and specificity for myocardial ischaemia of 78% and 52%, respectively, with a negative predictive value of 83%. A study of 5366 consecutive patients with suspected or established CAD who underwent stress electrocardiography-gated SPECT myocardial perfusion imaging demonstrated that inducible ischaemia identifies patients who benefit from short-term revascularization, while left ventricular ejection fraction (LVEF) predicts cardiac death. Similar to stress echocardiography, a normal stress SPECT MPI in patients with intermediate to high likelihood of CAD is associated with a very low rate of cardiac death or non-fatal MI (1%/year).^36^

The main limitations of SPECT remain 1/Lower spatial resolution compared to PET, particularly for evaluating small-calibre coronary arteries or patients with high body mass index (risk of artefacts).^37^ 2/The ionizing radiation exposure, with an average dose of approximately 10 mSv for protocols using Tc-99 m.

**Figure 5:**
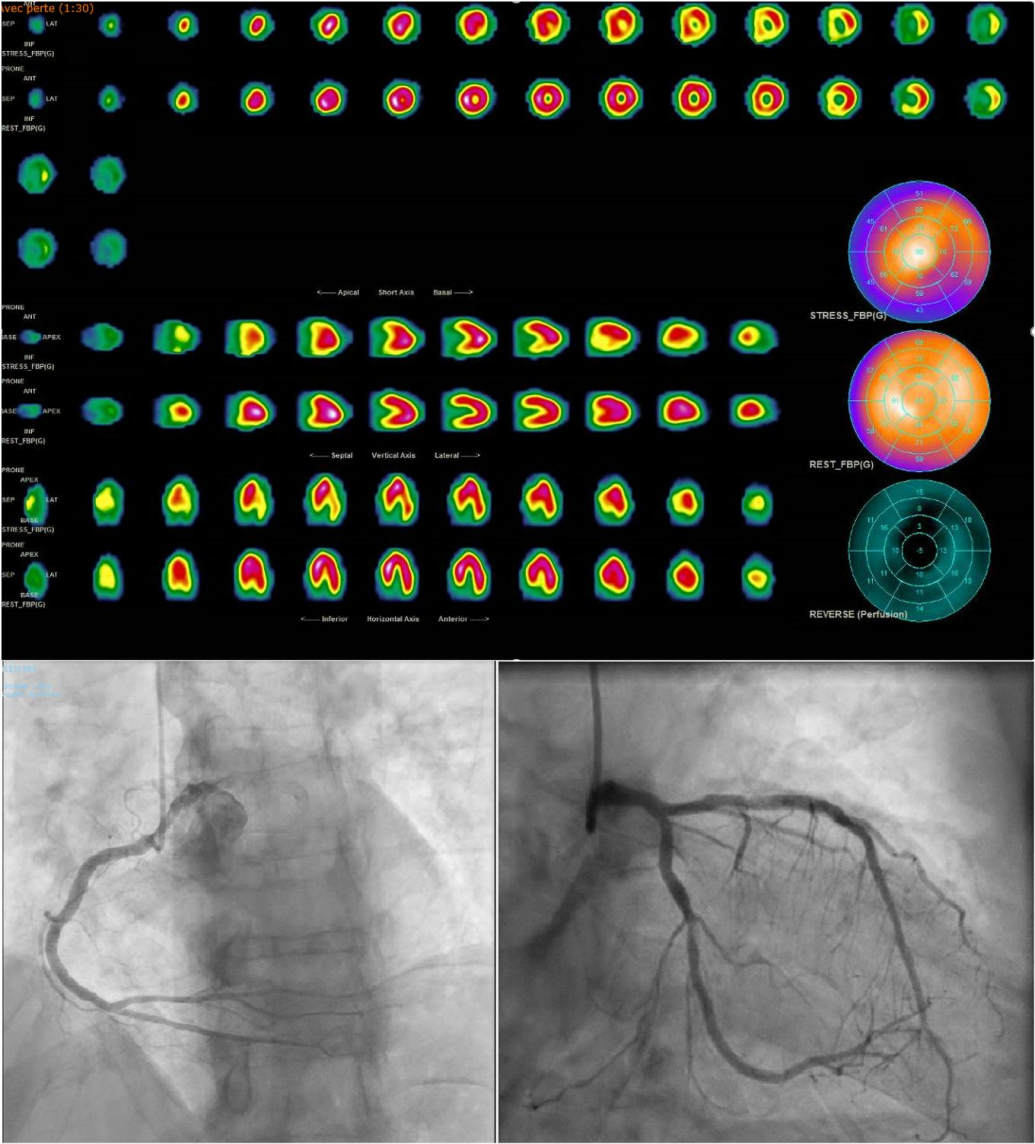
Myocardial perfusion imaging using SPECT in a 79-year-old post-LAD stent patient showing appearance of a reversible inferior and inferolateral ischaemia due to mid-circumflex stenosis, successfully treated by PCI.

We present the case of a 79-year-old man with hypertension and type 2 diabetes, previously stented in the LAD (2022), who presented with worsening exertional dyspnoea and chest discomfort. ECG was unremarkable. Myocardial perfusion imaging demonstrated completely reversible ischaemia in the inferior and inferolateral walls (*Figure 5*). Coronary angiography revealed a 70% mid-Cx stenosis, successfully treated with PCI, while a small ostial diagonal lesion was managed medically.

### Positron emission tomography (PET)

PET requires a non-contrast CT scanner for attenuation correction. It can be performed with catecholamine infusion (e.g. dobutamine) or vasodilators such as dipyridamole, adenosine, or Regadenoson. Usable radionuclides include 13N-ammonia, 15O-water, and 82Rubidium. McArdle *et al*.^38^ compared PET using rubidium-82 (Rb-82) to SPECT and found a sensitivity of 90% and specificity of 88% for detecting obstructive CAD. A perfusion defect ≥10% is prognostic, with each 10% increase of ischaemic myocardium, associated with a significant increase in cardiac death risk (from HR: 2.3 for mild ischaemia to 4.9 for severe ischaemia).^39,40^

Beyond better spatial resolution than SPECT, PET's advantage lies in measuring myocardial flow reserve (MFR), which reflects the increase in MBF achievable with maximal vasodilation induced by vasodilators. As the microvasculature primarily determines vascular resistance, MFR may enhance diagnostic and prognostic accuracy^41^ without extending imaging time. Patel *et al*.^42^ showed that MFR measured by PET is associated with all-cause mortality and identifies patients benefiting from early revascularization via PCI or CABG. A 0.1-unit decrease in MFR increases all-cause mortality risk by 9%. MFR predicts MACE more strongly than traditional risk factors or perfusion defects, especially in CCS patients.^43^

PET is particularly useful for coronary screening in obese patients (high photon energy), young patients (low radiation dose), and those with suspected diffusely impaired myocardial blood flow (MFR), such as in multivessel CAD or microvascular dysfunction.^44^ The non-contrast CT also required provides a CACS, offering additional information on coronary calcification (Agatston). Limitations include limited availability and challenges in performing physical stress testing in many patients.

### New guidelines for CCS imaging and future techniques

**Figure 6:**
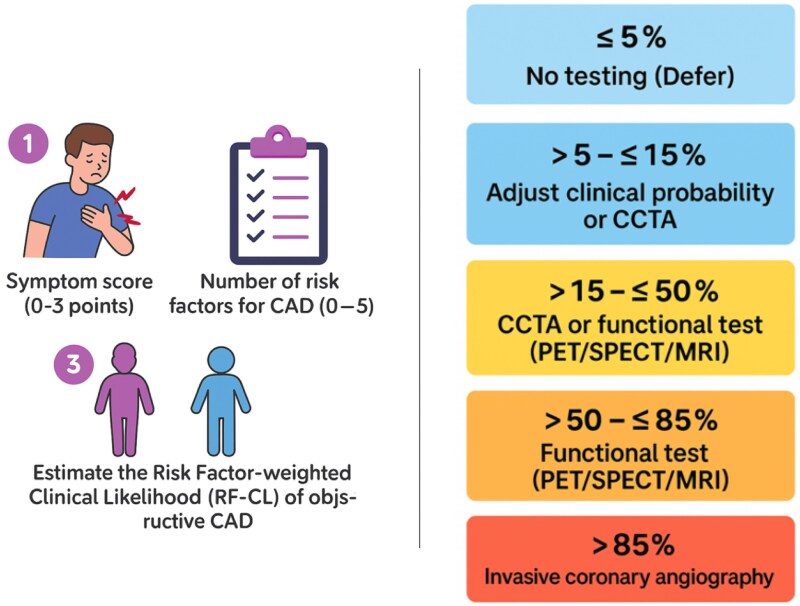
ESC proposed a diagnosis algorithm in suspected CCS according to pre-test probability.^1^

The 2019 ESC guidelines have defined an algorithm based on the advantages and limitations of each imaging modality and their validation by scientific evidence.^1^ Older clinical models were not discriminative enough to distinguish patients with non-obstructive lesions. It was therefore introduced the Risk Factor-weighted Clinical Likelihood (RF-CL) model^45^ to assess the pre-test probability of CCS. This model improves the identification of CCS patients. It uses a three-step approach: (i) A symptom score assigns 3 points for three chest pain characteristics, 2 points for two, and 0–1 for one or none, avoiding terms like ‘typical’ or ‘atypical’ angina; (ii) evaluates coronary artery disease risk factors; and (iii) calculates probability from the total score. Non-invasive imaging is then selected based on this probability (*Figure 6*), with invasive angiography reserved for high-probability cases. A multimodal approach combining anatomical and functional data enhances therapeutic decisions with priority for functional testing in MACE risk assessment.

**Figure 7:**
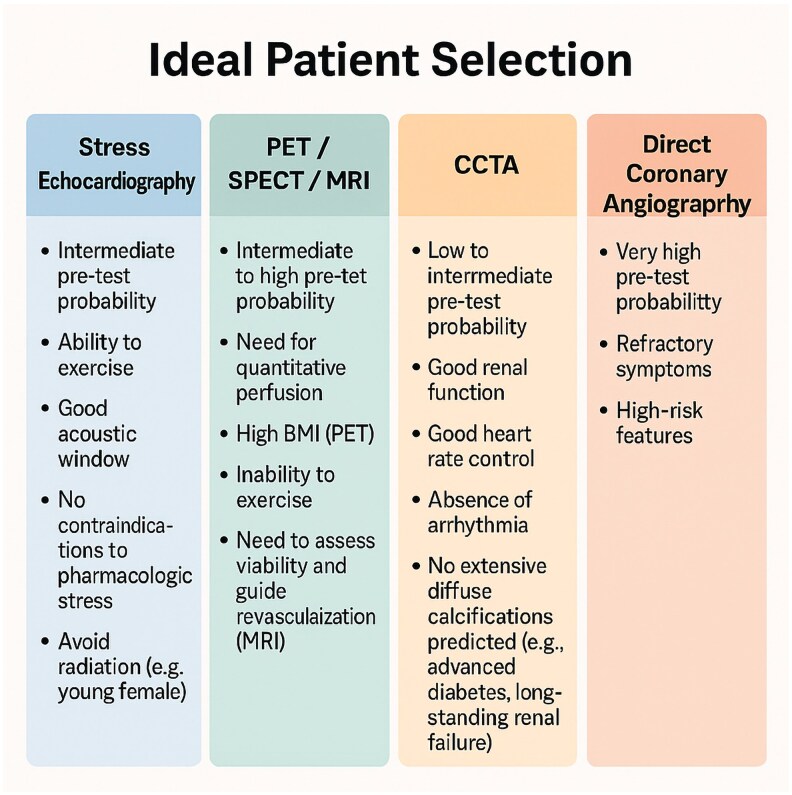
Ideal patient selection according to non-invasive imaging technique.

The AHA/ACC guidelines are similar,^46^ advocating an individualized approach. CCTA is primarily recommended for patients with low-to-intermediate pre-test probability and no known CAD due to its high negative predictive value. Functional tests (PET/SPECT MPI, stress cardiac MRI, stress echocardiography) are preferred for higher probabilities, established CAD, or significant calcifications. Mean cost in Europe per patient and each imaging comparison is displayed in *Table 2* while *Figure 7* summarizes the ideal patient selection for each technique.

**Table 2:** Comparison of non-invasive imaging for CCS

Modality	Sensitivity/Specificity	Key Strengths	Key Weaknesses	Mean Cost (Europe)
**CCTA**	95%/79–91%^47^	Anatomical detail high-risk plaque detection reduces invasive procedures (19–25%) cost-competitive (low-risk)	Calcification artefacts contrast risks heart rate issues	€200–400
**CMR**	81–86.5%/83.4–86%^28^	Non-irradiating, multiparametric (ischaemia, fibrosis) cost-effective prognostic value	Limited access Claustrophobia long acquisition prosthesis interference	€300–600
**SE**	85–95%/77–96%^24^	Detects ischaemia contractile reserve low cost non-invasive	Image quality operator-dependent variable accuracy	€140–400
**SPECT**	78%/52%^36^	Wide availability detects necrosis/ischaemia low event rate (1%/year)	Low resolution radiation (∼10 mSv) obese patient artefacts	€350–800
**PET**	90%/88%^39^	High accuracy MFR measurement suitable for obese/young low radiation	Limited availability high cost stress testing challenges	€700–1000

CCS: Chronic Coronary Syndrome; CCTA: Coronary Computed Tomography Angiography; CMR: Cardiovascular Magnetic Resonance; SE: Stress Echocardiography; SPECT: Single-Photon Emission Computed Tomography; PET: Positron Emission Tomography; MFR: Myocardial Flow Reserve

AI and advanced reconstruction techniques enhance CCTA by enabling rapid, automated quantification of coronary plaques (stenotic, calcified/non-calcified) and blood flow, validated against invasive standards for accurate anatomical and functional assessment.^48^ AI may enhance workflow, notably by calculating CT-derived fractional flow reserve (FFR CT), reducing invasive procedures, and improving risk stratification with combined functional and anatomical assessment. The ADVANCE study showed that FFR CT modifies treatment recommendations in 67% of cases compared to CCTA alone, reducing unnecessary invasive coronary angiographies (19% vs. 25%, *P* = 0.01) and predicting revascularizations without significant differences in costs or clinical outcomes, while other studies reported FFR CT’s 89% sensitivity and 76% specificity for detecting ischaemic lesions, potentially outperforming CCTA and SPECT in identifying ischaemia-causing lesions.^49,50^

Stress dynamic CT myocardial perfusion with CCTA offers comprehensive, non-invasive CAD evaluation, integrating anatomical and hemodynamic data, improving diagnostic accuracy (specificity: 35–88%, accuracy: 47–87%), and reducing unnecessary invasive procedures.^51^ It detects inducible ischaemia, guiding risk stratification and revascularization, with performance comparable to PET and stress MRI, surpassing SPECT.^52^

Current limitations include the need for high-quality images, technological complexity, limited availability, high costs, reimbursement challenges, and variable diagnostic accuracy in certain scenarios.

## Discussion

Advancements in imaging techniques associated with a better understanding of CAD have led to several outcomes:

First, the proliferation of non-invasive tests, especially when first-line tests are inconclusive or in asymptomatic patients, increases societal costs. For instance, the ESC estimated that 44% of patients with CCS^6,53^ do not follow the recommended diagnostic pathways, reflecting an increased use of these resources, even though the number of angiographies has not yet been shown to have increased at this stage.^54^

Second, the rationale for widespread screening of CCS is questioned, given the non-positive results of revascularization, as seen in ISCHAEMIA,^55^ notably for low-to-moderate ischaemic risk patients. At a population scale, these practices result in significant societal cost. Nevertheless, these techniques help to discriminate high-risk lesions and patients and avoid unnecessary invasive procedures for those without any lesions.

Third, CMR is the most cost-effective, has limited accessibility, PET offers high accuracy at a greater cost, and CCTA remains cost-competitive for low-risk patients. A systematic review confirms CMR cost-effectiveness of $19,273, compared to $19 578 for SPECT, $19 886 for CCTA, and $20 929 for invasive angiography.^56,57^

Finally, the prognosis improvement for robust clinical outcomes among patients with reduced LVEF (<35%) and revascularization has not yet been demonstrated compared to optimal therapy of heart failure.^58^ We can also mention the risk linked to cumulative X-ray exposure in increasingly younger patients (lifetime cancer risk ranges from 1 in 143 for a 20-year-old woman to 1 in 3261 for an 80-year-old man).^59^

This ‘imaging shift’ should lead to a reduction in purely diagnostic angiographies, though access to rapid functional tests, MRI, or CCTA remains challenging in some centres.

Non-invasive imaging will transform CCS management over the next decade, enabling precise diagnostics and prognostic stratification for personalized therapies. CMR represents the gold standard for functional analysis, myocardial viability, and tissue characterization, while CCTA excels in detecting atherosclerotic plaques and guiding revascularization when needed. However, increased reliance on these modalities may elevate societal costs, necessitating careful resource allocation.

## Learning points

1. CCTA, CMR, and PET enhance CCS diagnosis by providing complementary anatomical and functional insights at macro- and microvascular levels.
2. Multimodal imaging refines risk stratification and identification of high-risk patients, but raises concerns about cost, radiation exposure, and unproven outcome benefits in low-risk patients.
3. The 2019/2024 ESC guidelines propose a pre-test probability-based strategy, but 44% of CCS patients deviate from it—reflecting implementation gaps and unequal access to imaging.

## Supplementary Material

qyaf112_Supplementary_Data

## Data Availability

No new data were generated or analyzed in support of this research. The data discussed in this literature review are derived from previously published articles, accessible via the cited references. The clinical case examples provided are anonymized and do not include raw data.

## Lead author biography

David Sulman, MD is an Interventional Cardiologist at the Cardiology Institute, Pitié Salpêtrière, Paris, France, within the ACTION research group. He holds a medical degree from Université Paris Cité and a Master's Degree in Clinical Research from Université Sorbonne, specializing in cardiovascular disease and interventional cardiology. Dr. Sulman's research centers on coronary artery disease and cardiogenic shock.
